# Perceptions of coastal dwellers about the effects of extreme temperature and saline water on human health: evidence from Bangladesh

**DOI:** 10.3389/fpubh.2025.1451933

**Published:** 2025-08-01

**Authors:** Muhammad Zakaria, Rezaul Karim, Didar Islam, Md. Sarwar Ahmad, Mostak Ahammad, Feng Cheng, Junfang Xu

**Affiliations:** ^1^Department of Communication, Wayne State University, Detroit, MI, United States; ^2^Department of Communication and Journalism, University of Chittagong, Chattogram, Bangladesh; ^3^Department of Communication, Arizona State University, Tempe, AZ, United States; ^4^Department of Geography and Planning, University of Saskatchewan, Saskatoon, SK, Canada; ^5^Department of Mass Communication and Journalism, Begum Rokeya University, Rangpur, Bangladesh; ^6^Bangadesh Climate Change Journalists Forum (BCJF), Dhaka, Bangladesh; ^7^Vanke School of Public Health, Tsinghua University, Beijing, China; ^8^Institute for Healthy China, Tsinghua University, Beijing, China; ^9^School of Public Health, Zhejiang University School of Medicine, Hangzhou, China

**Keywords:** climate change, extreme temperature, water salinity, health effects, coastal area

## Abstract

**Background:**

Climate change disproportionately affects coastal communities worldwide, increasing exposure to extreme temperature and saline water intrusion. Understanding these impacts is critical for public health planning and intervention. This study aims to examine the perceptions about the effects of climate change-induced phenomena, specifically extreme temperature and water salinity, on the overall human health of coastal communities residing in Ramgati, Lakshmipur, Bangladesh.

**Methods:**

The study adopted a quantitative research approach and utilized a cross-sectional survey design to gather data. The sample consisted of 391 participants (*N* = 391) residing in the coastal region of Ramgati. A structured questionnaire was employed to collect data. The gathered data were subjected to several bivariate analyses, including independent-sample *t*-tests, Pearson correlation analysis, and hierarchical regression analysis, using IBM SPSS version 24.0.

**Results:**

Participants exposed to higher extreme temperature reported their perceptions of various health effects, such as heat stroke, dengue epidemic, migraine and headache. Additionally, participants experiencing elevated water salinity reported different health effects, including hair loss, high blood pressure, diarrhea, maternal health problems, child development, and hindered child mental health development. Linear regression analysis revealed that participants’ age (*β* = 0.33, *p* < 0.001), gender (*β* = −0.16, *p* < 0.001), perceived risk of health diseases (*β* = 0.17, *p* < 0.001), high salinity in water (*β* = 0.15, *p* = 0.002), and high temperature (*β* = 0.25, *p* < 0.001) were significantly associated with the perception of health effects.

**Conclusion:**

This study highlights the importance of addressing key issues regarding the effects of extreme temperature and saline water on human health. Specifically, the study reports on access to clean drinking water, climate change adaptation strategies, health education and awareness, an integrated public health approach, and the needs of vulnerable populations, in order to mitigate the effects of climate change on human health living in the coastal areas.

## Background

Climate change has emerged as a significant threat to humanity in recent years. It is characterized by global warming caused by human-generated greenhouse gas (GHG) emissions and the resultant large-scale alterations in weather patterns (1). Land temperatures are rising at nearly twice the global average, leading to the expansion of deserts and more frequent occurrences of heatwaves (1). Furthermore, increased evaporation rates contribute to the intensification of storms and extreme weather events (2). The Intergovernmental Panel on Climate Change (IPCC) has published a series of reports predicting substantial increases in these impacts as global temperatures rise to 1.5 degrees Celsius and beyond (1).

Climate change refers to long-term changes in the climate induced by natural or human-made forces that persist for decades or more (3). The variability in climate has significant impacts on public health, the economy, agriculture, water resources, and overall development of a country (4). For example, fluctuations in temperature and precipitation can lead to reduced agricultural productivity, while rising sea levels, storm surges, and extreme climatic events can damage or destroy coastal infrastructure, thus impeding socio-economic conditions. Additionally, the intrusion of seawater into land and aquifers poses threats to the agrarian sector, freshwater resources, and human health.

In Bangladesh, climate change is increasing the frequency and intensity of various weather-related disasters, while also heightening the vulnerability and undermining the resilience of communities that rely on arable land, access to water, and stable patterns of temperature and rainfall (5, 6). Climate change has the potential to amplify the risk of catastrophes by intensifying the occurrence and severity of hazard events, affecting vulnerability to such hazards, and altering exposure patterns, among other factors. Furthermore, due to its geographical location, socio-economic conditions, and physical characteristics, Bangladesh is highly susceptible to the impacts of climate change (5, 7). According to the United Nations Human Development report, Bangladesh ranks among the most vulnerable countries, with nearly 70 million people at risk from climate change (8), a majority of whom reside in the coastal regions.

The fourth assessment report of the IPCC explicitly states that climate change contributes to the global burden of disease and premature mortality (3). The relationship between climate variability and human health, however, is multifaceted, with both direct and indirect impacts intricately intertwined. Changing weather patterns directly affect individuals through factors such as variations in temperature and precipitation, sea-level rise, salinity intrusion, and more frequent extreme events. Indirectly, climate change affects human health through changes in water, air, and food quality, as well as alterations in ecosystems, agriculture, human settlements, and the economy (4, 9). These direct and indirect exposures have the potential to cause death, injury, and illness. For instance, climate change affects the seasonality of allergenic species and influences the seasonal activity and distribution of disease vectors like dengue and malaria. Furthermore, health issues can increase susceptibility and hinder people and organizations from effectively adapting to climate change (10, 11).

Climate change thus presents multifaceted threats to human health globally, including through extreme temperatures and saline water intrusion—two significant and interlinked phenomena. Rising global temperatures intensify heatwaves, exacerbating cardiovascular stress, heatstroke, and dehydration. Globally, extreme heat is responsible for substantial cardiovascular mortality, with approximately 93,000 heat-related cardiovascular deaths reported in 2019 alone (12). Bangladesh is particularly vulnerable, with heatwave events significantly increasing overall mortality rates (13).

Additionally, saline water intrusion resulting from sea-level rise poses significant health risks to coastal populations. In coastal Bangladesh, higher drinking-water salinity is linked to increased risks of hypertension and pregnancy-related complications, including preeclampsia (14, 15). Moreover, warmer temperatures and altered precipitation patterns due to climate change have extended the habitats and transmission seasons of disease vectors such as mosquitoes, contributing to outbreaks of vector-borne diseases like dengue fever (16, 17). Climate-induced nutritional impacts are also evident, as extreme weather events and flooding decrease agricultural productivity and food availability, exacerbating child undernutrition and stunting, particularly in vulnerable coastal areas (18, 19).

Thus, the interconnections between climate variability, extreme heat, salinity intrusion, and human health are clear and intensifying. Recognizing this nexus is crucial, especially for Bangladesh’s coastal communities that are increasingly at risk. Therefore, this study aims to examine coastal dwellers’ perceptions about health impacts arising from climate change-induced extreme temperatures and saline water exposure in Ramgati, Bangladesh.

## Methods

### Study design, study area and sample size

To achieve the desired research outcomes, this study employed a quantitative, cross-sectional design using a structured questionnaire survey method to collect primary data at a single point in time. The focus of the research was the coastal region of Bangladesh, which spans approximately 29,000 km^2^ and encompasses 19 districts, covering around 32% of the country’s land area (20). This region is highly vulnerable to various climate-induced phenomena and natural disasters, including cyclones, tidal floods, and seawater intrusion (10, 21). In recent years, the intensity and frequency of tropical cyclones, tidal actions, temperature fluctuations, and precipitation have been increasing, exacerbating the impact of high salinity levels in the coastal areas (10, 22, 23). It has been reported that 53% of the coastal area is affected by high salinity levels (24). As a result, local inhabitants in these areas are compelled to use saline water for their household needs, cooking, and drinking, leading to numerous health problems (5, 25).

The entire vast coastal region was not feasible for research. A specific area, Ramgati Upazila in Lakshmipur district, was chosen. Ramgati is located between 22°52′ and 22°90′ north latitudes and 90°47′ and 91°01′ east longitudes, at the estuary of the river Maghna and the Bay of Bengal. The area covers approximately 570.55 km^2^ with a population of 335,243. It includes eight unions and 50 mauzas with 39 villages. The region has a low literacy rate of 19.9% and depends mainly on agriculture for livelihood (66.56%). The primary sources of potable water for the local inhabitants are tube wells (75.84%), ponds (13.92%), and other sources (10.24%) (26).

The sample size for the study was calculated using the single population proportion formula, considering the following assumptions: a population proportion (*p*) of 50%, a significance level (*α*) of 5% (*α* = 0.05), a *Z*-value for a 95% confidence level (*Z α*/2) of 1.96, and a margin of error (*d*) of 3% (*d* = 0.05). However, we invited 400 respondents and collected data from 391 respondents with a response rate of 97.75%.

### Data collection process and instrument

Primary data were collected through a structured questionnaire survey. The questionnaire was developed considering local socio-economic contexts, relevant national and international guidelines, and existing literature regarding climate-induced phenomena and associated health impacts. The questionnaire included items addressing participants’ socio-economic and demographic characteristics (age, gender, occupation, family size, geographic location, educational level), commonly experienced climatic phenomena, disaster-related impacts, salinity levels, and associated health concerns. A Likert scale ranging from 1 to 5 was employed alongside multiple-choice questions for statement evaluation. Study participants, aged 18 years or older, were chosen using the convenient sampling method due to budget constraints. Prior to the survey, participants were informed about the study’s objective and confidentiality, and their consent was obtained.

### Content validity and reliability

For easy understanding, the questionnaire was translated into the local (Bengali) language from English content. After translation, it was revised by a language expert as the meaning and objectives of the question remain unchanged and easily understandable to local people. After that, the content of the questionnaire was evaluated by the subject expert panel to judge the scientific strength, nobility, and reliability of the research. The reliability was checked, and Cronbach’s Alpha (*α*) value of the 15 items related to the health effects is 0.72.

### Measurement

Most of the independent variables had nominal and ordinal measurements, which were recoded into dummies like education, employment status, gender, mobile use, internet use, risk perceptions of health disease, extreme temperature in the area, high level of salinity in the water and heavy rainfall in the area. However, each dummy variable was coded as 1 = Yes and 0 = No. Some covariates were continuous variables such as age and number of children. The study participants’ health effects were measured using 15 items. The 5-point Likert scale was used, ranging from 1 = ‘Never’, 2 = ‘Rarely’, 3 = ‘Occasionally’, 4 = ‘Frequently’ and 5 = ‘Very Frequently.’ The composite score of 15-items related to the perceptions of health effects was used as dependent variable for statistical analyses.

### Data analysis

The data were coded and entered into IBM SPSS version 24.0. An independent sample t-test was employed to evaluate differences in mean scores related to health outcomes influenced by variations in water salinity levels and extreme temperature conditions. Furthermore, to explore the bivariate relationships between health outcomes, demographic characteristics, and climate vulnerability, both independent-sample *t*-tests and Pearson correlation analyses were conducted. Variables showing a *p*-value less than 0.05 in the preliminary bivariate analyses were included in further regression modeling. In the regression analysis, variables with *p* < 0.05 were identified as significant predictors. The model summary indicates that the ANOVA values (*p* < 0.001) for each step of the hierarchical regression model indicate strong performance and predictive ability for the primary outcome variables. The *R^2^* values and *F* changes at each step exhibited substantial alterations and were statistically significant (*p* < 0.001). Variables showing a *p*-value below 0.05 in the regression analysis were considered significant predictors.

## Results

### Socio-demographic characteristics of study participants

Table 1 presents the socio-demographic characteristics of the participants involved in the study. A total of 391 individuals took part in the research. Among them, 91 (23.3%) had completed primary education, 57 (14.6%) had attained secondary education, 51 (13%) had completed higher secondary education, and 53 (13.6%) had obtained a degree in Honors/Bachelor. Regarding gender distribution, 105 participants (26.9%) were female, while 286 participants (73.1%) were male. The average age of the respondents was 38.17 (±12.93). In terms of media usage, 96 participants (24.6%) reported using media regularly, while 64 participants (16.4%) indicated that they never used media. Additionally, 99 participants (25.3%) reported using media rarely. Regarding technology usage, 276 participants (70.6%) reported using a mobile phone, while 155 participants (39.6%) reported using the internet.

**Table 1:** Socio-demographic background of study participants (*N* = 391).

Variables	Categories	Frequency	Percentage
Education	Illiterate	42	10.7
	Primary incomplete	64	16.4
	Primary	91	23.3
	Secondary	57	14.6
	Higher Secondary	51	13.0
	Honors/Degree	53	13.6
	Masters	33	8.4
Gender	Female	105	26.9
	Male	286	73.1
Age (M 38.17 years, SD 12.93)	Up to 30 years	123	31.5
	> 30–40 years	122	31.2
	> 40 years	146	37.3
Family size	Up to 5	92	23.5
	6–7	211	54.0
	> 7	88	22.5
Professional status	Housewife	61	15.6
	Agriculture/unemployment	58	14.8
	Lower employment	115	29.4
	Service/professional	157	40.2
Media use	Not at all	64	16.4
	Rare	99	25.3
	Now and then	56	14.3
	Often	76	19.4
	Regular	96	24.6
Mobile use	Yes	276	70.6
	No	115	29.4
Internet use	Yes	155	39.6
	No	236	60.4

### Descriptive statistics of the items related to the perceptions of health effects

Percentage and frequency distributions of the items related to the study participants’ perceptions of health effects caused by water salinity and extreme temperature are reported in Tables 2, 3. Of the study participants, 125 (32%) reported that they suffered from skin rash and skin allergy frequently (*M* = 2.98; 95% CI: 2.87–3.10), while 147 (37.6%) revealed that they had hair lose problem frequently caused by water salinity (*M* = 3.25; 95% CI: 3.15–3.36). Among the study participants, 107 (27.4%) had frequent blood pressure (*M* = 3.04; 95% CI: 2.92–3.16), whereas 93 (23.8%) had diabetes due to water salinity (*M* = 2.55; 95% CI: 2.42–2.68). Besides, of the study participants, 162 (41.4%) perceived that they sometimes suffered from acidity and gastrointestinal problems (*M* = 3.20; 95% CI: 3.10–3.31) and 139 (35.5%) sometimes suffered from diarrhea caused by water salinity (*M* = 3.08; 95% CI: 2.98–3.18). In addition, 47 (12.0%) had a perception of having frequent abortion problems (*M* = 2.09; 95% CI: 1.98–2.21) and 49 (12.5%) had a perception of having frequent maternal health problems (*M* = 2.24; 95% CI: 2.11–2.36). Moreover, of the study participants, 37 (9.5%) had a perception of having child development problems rarely due to water salinity (*M* = 1.72; 95% CI: 1.63–1.81) and 67 (17.1%) perceived that water salinity sometimes hindered child mental health. Regarding extreme temperature (*M* = 1.86; 95% CI: 1.77–1.96), 84 (21.5%) perceived that during summer high temperature sometimes caused heat stroke (*M* = 1.91; 95% CI: 1.81–2.00), and 96 (24.6%) perceived it caused dengue epidemic (*M* = 2.12; 95% CI: 2.01–2.22). Besides, 82 (21%) reported that they had a perception of high temperature that caused sometimes migraines and headaches (*M* = 1.98; 95% CI: 1.87–2.09), 141 (36.1%) for increasing pneumonia frequently (*M* = 3.52; 95% CI: 3.39–3.64) and 164 (41.9%) for causing cold and flu frequently (*M* = 4.11; 95% CI: 4.02–4.20).

**Table 2:** Frequency and percentage distributions of items regarding perceived health effects caused by extreme temperature and water salinity.

Items	Never	Rarely	Sometimes	Frequently	Very frequently
Water salinity causes skin rash and skin allergy	59 (15.1%)	69 (17.6%)	110 (28.1%)	125 (32.0%)	28 (7.2%)
Water salinity causes hair loss	33 (8.4%)	52 (13.3%)	124 (31.7%)	147 (37.6%)	35 (9.0%)
Water salinity causes high blood pressure	58 (14.8%)	73 (18.7%)	104 (26.6%)	107 (27.4%)	49 (12.5%)
Water salinity cause diabetes	106 (27.1%)	95 (24.3%)	93 (23.8%)	63 (16.1%)	34 (8.7%)
Water salinity causes acidity and gastrointestinal problems	31 (7.9%)	50 (12.8%)	162 (41.4%)	104 (26.6%)	44 (11.3%)
Water salinity cause diarrhea	24 (6.1%)	86 (22.0%)	139 (35.5%)	117 (29.9%)	25 (6.4%)
Water salinity causes abortion	168 (43.0%)	97 (24.8%)	63 (16.1%)	47 (12.0%)	16 (4.1%)
Water salinity causes problems in maternal health	146 (37.3%)	106 (27.1%)	65 (16.6%)	49 (12.5%)	25 (6.4%)
Water salinity causes child development problems	196 (50.1%)	138 (35.3%)	37 (9.5%)	11 (2.8%)	9 (2.3%)
Water salinity hinders child mental health	173 (44.2%)	130 (33.2%)	67 (17.1%)	11 (2.8%)	10 (2.6%)
High temperature during summer causes heat stroke	173 (44.2%)	112 (28.6%)	84 (21.5%)	14 (3.6%)	8 (2.0%)
High temperatures during summer cause dengue epidemic	136 (34.8%)	122 (31.2%)	96 (24.6%)	26 (6.6%)	11 (2.8%)
High temperature during summer causes migraine and headache	174 (44.5%)	98 (25.1%)	82 (21.0%)	27 (6.9%)	10 (2.6%)
Low temperature during winter increases pneumonia	50 (12.8%)	23 (5.9%)	85 (21.7%)	141 (36.1%)	92 (23.5%)
Low temperature during winter causes cold and flue	8 (2.0%)	11 (2.8%)	60 (15.3%)	164 (41.9%)	148 (37.9%)

**Table 3:** Descriptive statistics of different items relating to perceived health effects caused by extreme temperature and water salinity.

Items	*M*	SD	95% CI	Rank
Water salinity causes skin rash and skin allergy	2.98	1.18	2.87–3.10	8
Water salinity causes hair loss	3.25	1.07	3.15–3.36	3
Water salinity causes high blood pressure	3.04	1.25	2.92–3.16	7
Water salinity cause diabetes	2.55	1.28	2.42–2.68	9
Water salinity causes acidity and gastrointestinal problems	3.20	1.06	3.10–3.31	4
Water salinity cause diarrhea	3.08	1.01	2.98–3.18	6
Water salinity causes abortion	2.09	1.20	1.98–2.21	12
Water salinity causes problems in maternal health	2.24	1.25	2.11–2.36	10
Water salinity causes child development problems	1.72	0.92	1.63–1.81	16
Water salinity hinders child mental health	1.86	0.97	1.77–1.96	15
High temperature during summer causes heat stroke	1.91	0.99	1.81–2.00	14
High temperature during summer causes dengue epidemic	2.12	1.05	2.01–2.22	11
High temperature during summer causes migraine and headache	1.98	1.08	1.87–2.09	13
Low temperature during winter increases pneumonia	3.52	1.27	3.39–3.64	2
Low temperature during winter causes cold and flue	4.11	0.91	4.02–4.20	1

### Perceptions of health effects by extreme temperature and water salinity

Table 4 reveals that the study participants who had more extreme temperature perceived different types of health effects. An independent-sample t-tests show that the study participants with extreme temperature had perception of heat stroke (*t* = 6.56, *p* < 0.001), dengue epidemic (*t* = 10.41, *p* < 0.001), migraine and headache (*t* = 6.56, *p* < 0.001).

**Table 4:** Bivariate analysis of different items relating to the perceptions of health effects by exposure to extreme temperature.

Items	Exposed to extreme temperature		*t*	*p*
	Yes [*M* (*SD*)]	No [*M* (*SD*)]		
High temperature during summer causes heat stroke	2.18 (0.98)	1.56 (0.88)	6.56	<0.001
High temperature during summer causes dengue epidemic	2.55 (0.93)	1.57 (0.93)	10.41	<0.001
High temperature during summer causes migraine and headache	2.76 (0.84)	1.00 (0.00)	30.89	<0.001
Low temperature during winter increases pneumonia	3.62 (1.20)	3.39 (1.35)	1.82	0.070
Low temperature during winter causes cold and flue	4.18 (0.83)	4.02 (0.99)	1.66	0.099

Table 5 reveals that the study participants who had higher water salinity perceived different types of health effects. An independent-sample t-tests show that the study participants with extreme temperature had perception of hair loss (*t* = 3.74, *p* < 0.001), high blood pressure (*t* = 3.14, *p* = 0.002), diabetes (*t* = 3.00, *p* = 0.003), maternal health (*t* = 2.13, *p* = 0.033), child development problems (*t* = 6.03, *p* < 0.001), and hindering child mental health (*t* = 4.86, *p* < 0.001).

**Table 5:** Bivariate analysis of different items relating to the perceptions of health effects by the level of water salinity.

Items	Exposed to high water salinity		*t*	*p*
	Yes [*M* (*SD*)]	No [*M* (*SD*)]		
Water salinity causes skin rash and skin allergy	3.00 (1.26)	2.97 (1.07)	0.21	0.830
Water salinity causes hair loss	3.43 (1.00)	3.02 (1.12)	3.74	<0.001
Water salinity causes high blood pressure	3.21 (1.24)	2.82 (1.23)	3.14	0.002
Water salinity cause diabetes	2.71 (1.35)	2.33 (1.15)	3.00	0.003
Water salinity causes acidity and gastrointestinal problems	3.21 (1.10)	3.20 (1.01)	0.13	0.895
Water salinity cause diarrhea	2.96 (0.95)	3.26 (1.06)	−2.90	0.004
Water salinity causes abortion	2.17 (1.17)	2.00 (1.23)	1.36	0.175
Water salinity causes problems in maternal health	2.35 (1.17)	2.08 (1.34)	2.13	0.033
Water salinity causes child development problems	1.95 (0.93)	1.41 (0.81)	6.03	<0.001
Water salinity hinders child mental health	2.06 (0.95)	1.60 (0.94)	4.86	<0.001

### Hierarchical regression analysis for variables predicting study participants’ perceptions of health effects

The results of the hierarchical multiple regression analysis presented in Table 6 demonstrate the contribution of different predictors to participants’ perception of health effects. In step 1, participants’ age (*β* = 0.30, *t* = 6.36, *p* < 0.001) and gender (being male) (*β* = −0.21, *t* = −4.40, *p* < 0.001) significantly influenced the regression model (*F* = 30.11, *p* < 0.001) and accounted for 13% of the variance in the outcome variable.

**Table 6:** Linear regression depicting factors influencing study participants’ perceptions of health effects.

Variables	*R^2^*	Δ*R^2^*	*B*	*SE*	*β*	*t*	*p*
Step 1 (*F*_(2, 388)_ = 30.11, *p* < 0.001)	0.13	0.13	35.62	1.25		28.45	<0.001
Age^†^			0.17	0.03	0.30	6.36	<0.001
Being male^‡^			−3.52	0.80	−0.21	−4.40	<0.001
Step 2 (*F*_(3, 387)_ = 33.01, *p* < 0.001)	0.20	0.07	33.06	1.28		25.83	<0.001
Age^†^			0.19	0.03	0.33	7.13	<0.001
Being male^‡^			−3.14	0.77	−0.19	−4.06	<0.001
Perceived risk of health disease^‡^			4.00	0.69	0.27	5.81	<0.001
Step 3 (*F*_(4, 386)_ = 29.79, *p* < 0.001)	0.24	0.03	31.66	1.30		24.31	<0.001
Age^†^			0.19	0.03	0.33	7.31	<0.001
Being male^‡^			−2.95	0.76	−0.17	−3.89	<0.001
Perceived risk of health disease^‡^			3.02	0.72	0.20	4.21	<0.001
High salinity in water^‡^			2.88	0.72	0.19	4.03	<0.001
Step 4 (*F*_(5, 385)_ = 31.97, *p* < 0.001)	0.29	0.06	29.81	1.30		22.99	<0.001
Age^†^			0.19	0.03	0.33	7.69	<0.001
Being male^‡^			−2.62	0.73	−0.16	−3.58	<0.001
Perceived risk of health disease^‡^			2.59	0.70	0.17	3.73	<0.001
High salinity in water^‡^			2.23	0.70	0.15	3.20	0.002
High temperature^‡^			3.73	0.67	0.25	5.60	<0.001

^†^ Continuous variable; ^‡^ Dummy variable (1 = Yes, 0 = No).

Step 1: Δ*F* = 30.11; *df* (2, 388); *p* < 0.001; Step 2: Δ*F* = 33.73; *df* (1, 387); *p* < 0.001; Step 3: Δ*F* = 16.23; *df* (1, 386); *p* < 0.001; Step 4: Δ*F* = 31.35; *df* (2, 388); *p* < 0.001.

Moving to step 2, the inclusion of perceived risk of health disease as a predictor explained an additional 7% of the variation (*F* Change = 33.73, *p* < 0.001) in participants’ perception of health effects, beyond the effects of the predictors in step 1. In this model, participants’ age (*β* = 0.33, *t* = 7.13, *p* < 0.001), gender (*β* = −0.19, *t* = −4.06, *p* < 0.001), and perceived risk of health disease (*β* = 0.27, *t* = 5.81, *p* < 0.001) significantly contributed to the regression model (*F* = 33.01, *p* < 0.001), explaining a total of 20% of the variance in the outcome variable.

Progressing to step 3, the introduction of the high salinity in water factor alongside the other predictors accounted for an additional 3% of the variation (*F* Change = 16.23, *p* < 0.001) in participants’ perception of health effects, beyond the effects of the predictors in step 2. At this model, participants’ age (*β* = 0.33, *t* = 7.31, *p* < 0.001), gender (*β* = −0.17, *t* = −3.89, *p* < 0.001), perceived risk of health disease (*β* = 0.20, *t* = 4.21, *p* < 0.001), and high salinity in water (*β* = 0.19, *t* = 4.03, *p* < 0.001) significantly contributed to the regression model (*F* = 29.79, *p* < 0.001), accounting for a total of 23% of the variance in the outcome variable.

Moving on to step 4, the inclusion of the high temperature factor, in addition to the other predictors, explained an additional 6% of the variation (*F* Change = 31.35, *p* < 0.001) in participants’ perception of health effects, beyond the effects of the predictors in step 3. At this step, participants’ age (*β* = 0.33, *t* = 7.69, *p* < 0.001), gender (*β* = −0.16, *t* = −3.58, *p* < 0.001), perceived risk of health disease (*β* = 0.17, *t* = 3.73, *p* < 0.001), high salinity in water (*β* = 0.15, *t* = 3.20, *p* = 0.002), and high temperature (*β* = 0.25, *t* = 5.60, *p* < 0.001) significantly contributed to the regression model (*F* = 31.97, df = 5, *p* < 0.001), explaining a total of 28% of the variance in the outcome variable.

## Discussion

Our study found that coastal inhabitants perceived an association between extreme temperatures and migraines (*t* = 6.56, *p* < 0.001) and heat strokes (*t* = 6.56, *p* < 0.001), which is consistent with previous research (27–29). In hot and extreme temperatures, individuals are more susceptible to dehydration due to increased sweating and inadequate fluid intake. Dehydration can trigger migraines and headaches in vulnerable individuals. Furthermore, high temperatures, humidity, and intense sunlight act as triggers for migraines and increase the risk of heat stroke, particularly for those susceptible to environmental factors (30–32).

Additionally, this study reported that there was a perceived positive relationship between extreme temperatures and the spread of dengue epidemics (*t* = 10.41, *p* < 0.001). Our findings align with prior literature indicating that extreme temperatures indirectly contribute to the occurrence and spread of dengue epidemics in coastal communities (33–36). Warmer temperatures create favorable conditions for mosquitoes, especially the *Aedes aegypti* mosquito, the primary vector for dengue fever. Mosquitoes exhibit increased activity and accelerated breeding and reproduction rates in warm weather. As extreme temperatures can lead to water scarcity in coastal areas, communities may store water in containers or tanks. However, if these storage practices are not properly covered or maintained, they inadvertently create breeding grounds for mosquitoes.

Our study participants perceived that there is a significant association between water salinity and hair loss (*t* = 3.74, *p* < 0.001), hypertension (*t* = 3.14, *p* = 0.002), and diabetes (*t* = 3.00, *p* = 0.003) among coastal communities in Ramgati Upazila, Bangladesh. Existing literature supports the notion that saltwater can strip away natural scalp oils, leading to dry and brittle hair follicles, which can contribute to hair loss (37). Exposure to high salinity water weakens hair follicles, making them more susceptible to breakage and loss. Saltwater can also cause scalp inflammation, leading to conditions like scalp dermatitis, which further contribute to hair loss.

Water salinity, particularly in areas with high salt content, can also increase sodium intake through drinking water and food. Excessive sodium consumption disrupts fluid balance, causing fluid retention and increased blood volume, ultimately leading to hypertension. Limited availability of freshwater sources due to water salinity pushes communities to rely on brackish water, reducing access to safe drinking water and increasing the risk of diabetes (38, 39). Saline water can also impact agriculture and crop yields, resulting in reduced availability of fresh and nutritious foods, further increasing the risk of diabetes (40, 41).

Furthermore, the study participants perceived that water salinity can affect reproductive health and fertility (*t* = 2.13, *p* = 0.033). |Our findings support previous literature indicating that water salinity can affect reproductive health and fertility (37, 42, 43). Excessive salt intake or exposure to high salinity water can disrupt hormonal balance and menstrual regularity, potentially leading to fertility problems. During pregnancy, consuming water with high salinity can cause complications like pre-eclampsia, characterized by hypertension and organ damage, posing risks to both the mother and the fetus.

The study participants perceived that water salinity hinders child development (*t* = 6.03, *p* < 0.001) and mental health growth (*t* = 4.86, *p* < 0.001), which is similar with the findings of previous research (42–44). Water salinity’s impact on agriculture and reduced crop yields limits coastal communities’ access to nutrient-rich foods, including fresh fruits and vegetables. Malnutrition and inadequate nutrient intake negatively affect cognitive and physical growth in children. Water salinity can also lead to micronutrient deficiencies, such as iodine or iron deficiencies, crucial for proper brain development and cognitive function in children.

The study on the impact of water salinity and extreme temperatures on the health of coastal communities in Ramgati Upazila, Lakshmipur, Bangladesh suggests significant policy interventions. To mitigate these effects, policies should ensure access to clean drinking water through desalination, water purification systems, and establishing alternative freshwater sources, alongside rigorous water quality monitoring and regulations. Additionally, comprehensive climate change adaptation strategies are crucial. These should include infrastructure enhancements, early warning systems, and community-based resilience initiatives integrated into policy frameworks and urban planning to facilitate long-term adaptation and sustainable development.

### Limitations and future research

The study has several limitations that need to be acknowledged. First, the research was conducted solely in Ramgati Upazila of Lakshmipur, focusing on a single coastal area. Results from a single location (Ramgati Upazila) may not apply directly to other coastal regions, as different areas might have distinct environmental conditions, resources, infrastructure, and socio-economic characteristics. This study suggests conducting comparative studies in multiple coastal regions (e.g., Satkhira, Barguna, Cox’s Bazar) in the future to strengthen external validity and generalizability. Future research across multiple coastal sites would enhance the robustness of findings and improve policy applicability.

Second, due to budgetary constraints, the selection of study participants relied on convenient sampling techniques. This sampling method may introduce selection bias and raise questions regarding the generalizability of the findings to the broader population. Future research should consider employing random or stratified sampling techniques to enhance representativeness and reduce bias.

Third, the data regarding health effects were gathered through self-reported perceptions of the study participants, rather than relying on medical records or objective measurements. This reliance on subjective perceptions may introduce biases or inaccuracies into the findings. Participants might inaccurately report symptoms, over- or underestimate the severity of health problems, or provide socially acceptable responses rather than accurate descriptions. This study suggests complementing self-reported data with objective data sources (e.g., medical records, clinical measurements, lab tests) in future research.

Fourth, due to its cross-sectional design, it is challenging to establish causal relationships between extreme temperature, saline water, and perceived health outcomes. The study provides a snapshot of the situation at a specific point in time, making it difficult to determine the cause-and-effect dynamics accurately. Future research employing longitudinal designs would help clarify temporal and causal relationships.

Fifth, the study may not have captured the perceptions of all possible health outcomes. This study suggests including a broader range of health indicators, including mental health and chronic diseases, complemented by qualitative assessments.

Lastly, confounding variables such as socio-economic status, healthcare accessibility, and pre-existing medical conditions were inadequately controlled. Future research should explicitly measure and statistically control these variables for stronger validity.

It is important to consider these limitations when interpreting the results of the study and to recognize the potential impact they may have on the generalizability and validity of the findings.

## Conclusion

Water salinity and extreme temperature have been found to have significant health effects among coastal communities residing in Ramgati Upazila, Lakshmipur, Bangladesh. The identified health effects include hair loss, high blood pressure, diabetes, maternal health issues, child development problems, hindered child mental health, dengue epidemics, heat stroke, migraines and headaches, as well as cold and flu. Furthermore, the study revealed that age, gender (being female), and perceived risk of health diseases were significant predictors of perceived health effects among the coastal community residents.

These findings emphasize the urgent need for targeted actions by specific institutions and organizations in Bangladesh to address health implications associated with extreme temperatures in coastal regions. The Ministry of Health and Family Welfare, and Ministry of Water Resources, Bangladesh, in collaboration with local government bodies (such as Union Parishads and Upazila Parishads), should prioritize improving access to clean drinking water and developing comprehensive climate change adaptation strategies tailored to local environmental conditions. Non-governmental organizations (NGOs) must actively engage in health education and awareness campaigns, focusing on preventive measures against heat-related illnesses. Additionally, the Directorate General of Health Services (DGHS) should integrate climate health concerns into existing public health frameworks, ensuring equitable healthcare access for vulnerable populations such as women, children, the older adults, and socioeconomically disadvantaged groups. By addressing these pressing issues, policymakers and stakeholders can work toward safeguarding the health and well-being of coastal communities, reducing the burden of disease, and promoting resilience in the face of environmental challenges.

## Data Availability

The raw data supporting the conclusions of this article will be made available by the authors, without undue reservation.

## References

[1] Intergovernmental Panel on Climate Change. (2019). Climate change and land: an IPCC special report on climate change, desertification, land degradation, sustainable land management, food security, and greenhouse gas fluxes in terrestrial ecosystems. Available online at: https://www.ipcc.ch/site/assets/uploads/2019/11/SRCCL-Full-Report-Compiled-191128.pdf (Accessed June 6, 2021).

[2] Islam A Majumder A. Hypertension in Bangladesh: a review. Indian Heart J. (2012) 6403:319–23. doi: 10.1016/S0019-4832(12)60096-0 PMC3860599 22664819

[3] Intergovernmental Panel on Climate Change (IPCC). Fourth assessment summary report 2007. Kenya: UNEP/UNDP (2007).

[4] Shahid S. Probable impacts of climate change on public health in Bangladesh. Asia Pac J Public Health. (2010) 22:310–9. doi: 10.1177/1010539509335499, PMID: 19443872

[5] Islam SMD Majumder RK Uddin MJ Khalil MI Alam MF. Hydrochemical characteristics and quality assessment of groundwater in Patuakhali district, southern coastal region of Bangladesh. Expo Health. (2017) 9:43–60. doi: 10.1007/s12403-016-0221-y

[6] Rahman MS Islam ARMT. Are precipitation concentration and intensity changing in Bangladesh overtimes? Analysis of the possible causes of changes in precipitation systems. Sci Total Environ. (2019) 690:370–87. doi: 10.1016/j.scitotenv.2019.06.529, PMID: 31299571

[7] Rahman MM Bodrud-Doza M Shammi M Islam ARMT Khan ASM. COVID-19 pandemic, dengue epidemic, and climate change vulnerability in Bangladesh: scenario assessment for strategic management and policy implications. Environ Res. (2021) 192:110303. doi: 10.1016/j.envres.2020.110303, PMID: 33069704 PMC7561529

[8] United Nations Development program. Human Development report 2007.08: fighting climate change-human solidarity in a divided world. New York, USA: UNDP (2007).

[9] Rahman A. Climate change and its impact on health in Bangladesh. Reg Health Forum. (2008) 12:16–26.

[10] Azam G Huda ME Bhuiyan MAH Mohinuzzaman M Bodrud-Doza M Islam SMD. Climate change and natural hazards vulnerability of char land (bar land) communities of Bangladesh: application of the livelihood vulnerability index (LVI). Glob Soc Welf. (2019) 8:1. doi: 10.1007/s40609-019-00148-133738179

[11] Islam SMD Bhuiyan MAH. Exploring climate change adaptation in coastal communities of Bangladesh using adaptation capability index (ACI). Jahangirnagar Univ Environ Bull. (2016) 5:11–23.

[12] Le H Yan MM Zhang YQ Wang K Wang YQ Luo SQ. Global burden of cardiovascular disease attributable to high temperature in 204 countries and territories from 1990 to 2019. Biomed Environ Sci. (2023) 36:222–30. doi: 10.3967/bes2023.025 37005076

[13] Nissan H Burkart K Coughlan de Perez E van Aalst M Mason SJ. Defining and predicting heat waves in Bangladesh. J Appl Meteorol Climatol. (2017) 56:2653–70. doi: 10.1175/JAMC-D-17-0035.1

[14] Khan AE Scheelbeek PFD Shilpi AB Chan Q Mojumder SK Rahman A. Salinity in drinking water and risk of (pre)eclampsia and gestational hypertension in coastal Bangladesh: a case-control study. PLoS One. (2014) 9:e108715. doi: 10.1371/journal.pone.0108715 25268785 PMC4182542

[15] Talukder MRR Rutherford S Phung D Islam MZ Chu C. The effect of drinking water salinity on blood pressure in young adults of coastal Bangladesh. Environ Pollut. (2016) 214:248–54. doi: 10.1016/j.envpol.2016.03.074, PMID: 27089422

[16] Caminade C McIntyre KM Jones AE. Impact of recent and future climate change on vector-borne diseases. Ann N Y Acad Sci. (2019) 1436:157–73. doi: 10.1111/nyas.13950, PMID: 30120891 PMC6378404

[17] Mahmud AS Bhattacharjee J Baker RE Martinez PP. Alarming trends in dengue incidence and mortality in Bangladesh. J Infect Dis. (2024) 229:4–6. doi: 10.1093/infdis/jiad529, PMID: 38000901 PMC10786241

[18] McMahon K Gray C. Climate change, social vulnerability and child nutrition in South Asia. Glob Environ Change. (2021) 71:102414. doi: 10.1016/j.gloenvcha.2021.102414, PMID: 34898861 PMC8653856

[19] Rahman M Sarkar P Islam MJ Adam IF Nguyen NHC Al-Sobaihi S. Factors mediating the association between recurring floods and child chronic undernutrition in northern Bangladesh. Nutrition. (2024) 119:112300. doi: 10.1016/j.nut.2023.112300 38141569

[20] MoWR. Coastal zone policy (CZPo) Ministry of Water Resources (MoWR), government of the people’s republic of Bangladesh. Dhaka: Ministry of Water Resources (2005).

[21] Bernard A Long N Becker M Khan J Fanchette S. Bangladesh’s vulnerability to cyclonic coastal flooding. Nat Hazards Earth Syst Sci. (2022) 22:729–51. doi: 10.5194/nhess-22-729-2022

[22] Arif MSI Mahdi I Rafi MA Khan SJ Rahman MM. Cyclone exposure mapping in coastal Bangladesh: a multi-criteria decision analysis. Heliyon. (2023) 9:e21259. doi: 10.1016/j.heliyon.2023.e21259, PMID: 37928379 PMC10622688

[23] Dasgupta S Hossain MM Huq M Wheeler D. Climate change and soil salinity: the case of coastal Bangladesh. Ambio. (2015) 44:815–26. doi: 10.1007/s13280-015-0681-5, PMID: 26152508 PMC4646857

[24] Hoque M Hasan MK Ravenscroft P. Investigation of groundwater salinity and gas problems in Southeast Bangladesh In: Rahman AA Ravenscroft P, editors. Groundwater resources and development in Bangladesh. Dhaka: Bangladesh Centre for Advanced Studies, University Press Ltd. (2003)

[25] Mustari S Karim AHMZ. Impact of salinity on the socio-environmental life of coastal people of Bangladesh. Asian J Soc Sci Humanit. (2014) 3:12–8.

[26] Bangladesh Bureau of Statistics (BBS). Population and housing Census-2011, community report, Lakshmipur zila. Dhaka: Ministry of Planning, Government of Bangladesh (2011).

[27] Amiri M Peinkhofer C Othman MH De Vecchi T Nersesjan V Kondziella D. Global warming and neurological practice: systematic review. PeerJ. (2021) 9:e11941. doi: 10.7717/peerj.11941, PMID: 34430087 PMC8349167

[28] Louis S Carlson AK Suresh A Rim J Mays M Ontaneda D. Impacts of climate change and air pollution on neurologic health, disease, and practice: a scoping review. Neurology. (2023) 100:474–83. doi: 10.1212/WNL.0000000000201630, PMID: 36384657 PMC9990849

[29] Luber G McGeehin M. Climate change and extreme heat events. Am J Prev Med. (2008) 35:429–35. doi: 10.1016/j.amepre.2008.08.021, PMID: 18929969

[30] Li G Guo Q Liu Y Li Y Pan X. Projected temperature-related years of life lost from stroke due to global warming in a temperate climate city, Asia: disease burden caused by future climate change. Stroke. (2018) 49:828–34. doi: 10.1161/STROKEAHA.117.020042, PMID: 29523649

[31] Martinez GS Imai C Masumo K. Local heat stroke prevention plans in Japan: characteristics and elements for public health adaptation to climate change. Int J Environ Res Public Health. (2011) 8:4563–81. doi: 10.3390/ijerph8124563, PMID: 22408589 PMC3290973

[32] Tewari K Tewari M Niyogi D. Need for considering urban climate change factors on stroke, neurodegenerative diseases, and mood disorders studies. Comput Urban Sci. (2023) 3:4. doi: 10.1007/s43762-023-00079-w

[33] Barclay E. Is climate change affecting dengue in the Americas? Lancet. (2008) 371:973–4. doi: 10.1016/S0140-6736(08)60435-3, PMID: 18363202

[34] Misslin R Telle O Daudé E Vaguet A Paul RE. Urban climate versus global climate change—what makes the difference for dengue? Ann N Y Acad Sci. (2016) 1382:56–72. doi: 10.1111/nyas.13084, PMID: 27197685

[35] Naish S Dale P Mackenzie JS McBride J Mengersen K Tong S. Climate change and dengue: a critical and systematic review of quantitative modelling approaches. BMC Infect Dis. (2014) 14:1–14. doi: 10.1186/1471-2334-14-167, PMID: 24669859 PMC3986908

[36] Robert MA Christofferson RC Weber PD Wearing HJ. Temperature impacts on dengue emergence in the United States: investigating the role of seasonality and climate change. Epidemics. (2019) 28:100344. doi: 10.1016/j.epidem.2019.05.003, PMID: 31175008 PMC6791375

[37] Paul A Jabed MA. Salinity has made our life terrible: a qualitative investigation of human sufferings in the Chittagong coast. Orient Geogr. (2018) 59:1–18.

[38] Natur S Damri O Agam G. The effect of global warming on complex disorders (mental disorders, primary hypertension, and type 2 diabetes). Int J Environ Res Public Health. (2022) 19:9398. doi: 10.3390/ijerph19159398, PMID: 35954764 PMC9368177

[39] Wang X Li G Liu L Westerdahl D Jin X Pan X. Effects of extreme temperatures on cause-specific cardiovascular mortality in China. Int J Environ Res Public Health. (2015) 12:16136–56. doi: 10.3390/ijerph121215042, PMID: 26703637 PMC4690978

[40] He FJ MacGregor GA. A comprehensive review on salt and health and current experience of worldwide salt reduction programmes. J Hum Hypertens. (2009) 23:363–84. doi: 10.1038/jhh.2008.144, PMID: 19110538

[41] Khan A Ireson A Kovats S Mojumder S Khusru A Rahman A. Drinking water salinity and maternal health in coastal Bangladesh: implications of climate change. Environ Health Perspect. (2011) 119:1328–32. doi: 10.1289/ehp.1002804, PMID: 21486720 PMC3230389

[42] Saha SK. Cyclone, salinity intrusion and adaptation and coping measures in coastal Bangladesh. Space Cult India. (2017) 5:12–24. doi: 10.20896/saci.v5i1.234

[43] Warner K. Van der Geest K. Huq S. Harmeling S. Kusters K. de Sherbinin A.. (2012) Evidence from the frontlines of climate change: loss and damage to communities despite coping and adaptation United Nations University-Institute for Environment and Human Security (UNU-EHS). Available online at: http://loss-anddamage.net/download/6815.pdf (Accessed October 12, 2023).

[44] Paul B Rashid H. Climatic hazards in coastal Bangladesh: non-structural and structural solutions. Oxford, UK: Butterworth-Heinemann (2016).

